# The Value of Gonococcic Vaccine in the Treatment of Gonorrheal Arthritis

**Published:** 1910-05

**Authors:** W. L. Champion

**Affiliations:** Atlanta, Ga.


### THE VALUE OF GONOCOCCIC VACCINE IN THE TREATMENT OF GONORRHEAL ARTHRITIS.

BY W. L. CHAMPION, M. D., ATLANTA, GA.

This will be a short paper to give the results obtained in my* private work from the use of gonococcic "Vaccine" or "Bacteria." Before the introduction of the vaccine and serum there was not a drug to be relied upon to cure or I might say benefit a patient with gonorrheal rheumatism, so I hailed with delight this new remedy when I noticed the favorable reports from its use. The invasion of the joints and muscles by the gonococcus and its toxins is one of the most serious complications that follow an attack of gonorrhea. It is not unusual to see a patient confined to his bed for weeks and months and his joints permanently ankylosed.

I recall the case of a young man I treated several years ago who was rendered helpless for three months by the involvement of a knee, ankle, toe, elbow, wrist and finger joints, and finally it was necessary to open and drain the knee to make it useful. Such results I have seen before the introduction of gonococcic vaccine and anti-gonococcic serum. Since I began the use of these remedies I have had a number of patients to suddenly develop rheumatism of a single joint with severe pain, swelling,

*Read at Medical Association of Georgia, Athens, April 18 to 20, 1910.

temperature reach 101 to 103, and a single injection of the vaccine make the patient comfortable in a few hours, and five or six injections perfect a cure.

The above results with the vaccine have convinced me that it is a specific in acute gonorrheal rheumatism. While the percentage of gonococcics that develop this complication is apparently small, I am convinced if we will listen attentively to the complaints made by these patients, of pains and soreness located in various muscles and joints, we will be convinced it is more frequent than stated. The attack may be of a mild form, lasting only a few days; the patient and doctor attributing the condition to cold or other causes when nature comes to the rescue, and the fact that gonorrheal poison has entered the circulation from the deep urethra, prostate or vesicles is never recorded.

My work is confined almost exclusively to the male side of the human family, but of the few women I have treated with gonorrhea I have not recorded a single case of gonorrheal rheumatism. This may be accounted for from the fact that female has no prostate, as every case I have seen in the male the prostate was involved. The prostate seems to be open to engagements to send the septic material it contains through the general circulation.

During the past twenty months I have treated eleven typical cases of gonorrheal rheumatism with Mulford's gonococcic vaccine. All of the patients had severe pain, redness, swelling of the joint, elevation of temperature and rapid pulse. It is very noticeable that the pulse is rapid; out of proportion to the temperature, and remains rapid after the temperature returns to normal. In all of these cases the vaccine was used in the beginning of the complication, and had not been used previously. None of the patients had other joints to become involved after beginning the use of the vaccine.

Ten of the patients were under thirty years of age, the other one forty. Of the eleven cases three had a single joint involved; two had both knees; one the right foot, and the other five had three to four joints involved. Only two of the eleven cases were confined to bed; one for six days and the other for ten. In every case there was marked improvement from the first dose of the vaccine.

The dose I commence with is 25,000,000 bacteria, giving 50,000,000 on the third day, and a similar dose every four days thereafter.

In conclusion, I would urge the use of the vacine as soon as there is the slightest evidence of rheumatism developing, as it will shorten the course of the disease and lessen the number of joints involved.

313 and 314 Grant Building.

